# Cerium Oxide Nanoparticles Induced Toxicity in Human Lung Cells: Role of ROS Mediated DNA Damage and Apoptosis

**DOI:** 10.1155/2014/891934

**Published:** 2014-06-01

**Authors:** Sandeep Mittal, Alok K. Pandey

**Affiliations:** ^1^Academy of Scientific and Innovative Research (AcSIR), New Delhi 110025, India; ^2^Nanomaterial Toxicology Group, CSIR-Indian Institute of Toxicology Research (CSIR-IITR), P.O. Box 80, Mahatma Gandhi Marg, Lucknow, Uttar Pradesh 226001, India

## Abstract

Cerium oxide nanoparticles (CeO_2_ NPs) have promising industrial and biomedical applications. In spite of their applications, the toxicity of these NPs in biological/physiological environment is a major concern. Present study aimed to understand the molecular mechanism underlying the toxicity of CeO_2_ NPs on lung adenocarcinoma (A549) cells. After internalization, CeO_2_ NPs caused significant cytotoxicity and morphological changes in A549 cells. Further, the cell death was found to be apoptotic as shown by loss in mitochondrial membrane potential and increase in annexin-V positive cells and confirmed by immunoblot analysis of BAX, BCl-2, Cyt C, AIF, caspase-3, and caspase-9. A significant increase in oxidative DNA damage was found which was confirmed by phosphorylation of p53 gene and presence of cleaved poly ADP ribose polymerase (PARP). This damage could be attributed to increased production of reactive oxygen species (ROS) with concomitant decrease in antioxidant “glutathione (GSH)” level. DNA damage and cell death were attenuated by the application of ROS and apoptosis inhibitors N-acetyl-L- cysteine (NAC) and Z-DEVD-fmk, respectively. Our study concludes that ROS mediated DNA damage and cell cycle arrest play a major role in CeO_2_ NPs induced apoptotic cell death in A549 cells. Apart from beneficial applications, these NPs also impart potential harmful effects which should be properly evaluated prior to their use.

## 1. Introduction

Over the past few years, there has been rapid increase in the use of different nanomaterials owing to their unique physicochemical and bioreactive properties. Different metal oxide nanoparticles (NPs) have potentially been used in industries including sunscreens, food, paints, textile, electronics, sports, and biomedical application and imaging [1, 2]. It is estimated that the value of engineered nanoparticles (ENPs) market would increase up to 20–30 billion dollars by 2015 [3]. This has raised concerns over the unforeseen adverse harmful health effects caused due to interactions with the living systems.

Amongst rare earth elements, cerium oxide (CeO_2_) NPs are widely used in a variety of applications such as glass/ceramic polishing agent, television tubes, solar cells, fuel cells, ultraviolet absorbents, and gas sensors [4–7]. Besides these industrial applications, various biomedical applications of CeO_2_ NPs such as protection against radiation induced damage and retinal neurodegeneration and anti-inflammatory and antioxidant activity have also been explored [8–11]. Recently the use of CeO_2_ NPs as a diesel fuel additive, to reduce the ignition temperature of carbonaceous diesel exhaust particle (DEP) and subsequently to reduce the emission of particulate matter from diesel engines, has been explored [12]. Although this addition enhances the ability of diesel engines, it leads to direct emission of CeO_2_ NPs in the environment. Health Effect Institute (HEI) has also reported that the CeO_2_ NPs emission will reach up to 22 million pounds annually in European Union after this addition [12]. Thus, commercially used CeO_2_ NPs are released into the environment and their evaluation in the living system is worthwhile and relevant for society and human welfare [13]. Human exposure to the nanoparticles is possible both from workplace (occupational) and environmental release through inhalation and ingestion as major routes. Since CeO_2_ NPs are poorly absorbed by the intestine, inhalation appears to be the major route of exposure. It should also be noted that complete respiratory system acts as repository for deposition of different sizes of NPs. Although several studies have been performed to evaluate the adverse effect of CeO_2_ NPs on environment and human health, they are not providing proper conclusion. CeO_2_ NPs have been reported to act as cellular antioxidants with colocalizations inside mitochondria, lysosomes, endoplasmic reticulum, nucleus, and cytoplasm using keratinocyte model systems [14, 15]. However, the study lacked genotoxicity assessment of these particles. Contrary to this, previous studies have reported that CeO_2_ NPs generate oxidative stress and may induce apoptosis in human lung epithelial cells [16, 17]. Therefore, CeO_2_ NPs may portend cytotoxic and genotoxic effects upon cellular internalization. It has been reported that the CeO_2_ NPs of various sizes showed significant toxic effects on* E. coli* and human cells, respectively, due to adsorption of NPs and oxidative stress [18, 19]. Along this, reports have also been postulated that CeO_2_ NPs of smaller sizes do not cause any adverse effect but can protect cells from harmful effects of radiation and oxidative stress, although this protection was cell type specific [20, 21].* In vivo* studies have resulted that CeO_2_ NPs exposure via inhalation and intratracheal instillation route can induce acute pulmonary and systemic toxicity in rat and mice due to proinflammatory responses [22, 23]. Besides these, CeO_2_ NPs have been shown to protect cells from reactive oxygen species due to their inherent antioxidant properties [15, 24]; this protective effect was thought to be due to the presence of dual oxidation state of CeO_2_ NPs or the pH value of cell compartment where the nanoparticles internalize. With these contrasting results, the toxicity of CeO_2_ NPs remains elusive and specific toxicity endpoints relevant to human health needs to be addressed. It was therefore prudent to conduct a systematic study to understand the comprehensive molecular mechanism of toxicity of CeO_2_ NPs as well as to see the role of DNA damage and halt of cell cycle in cell death.

Characterization of NPs is an essential step before toxicity assessment as properties of NPs vary significantly with shape and size. It is also essential to characterize their agglomeration/aggregation tendency in the culture media for accurate toxicity assessment.* In vitro* cell based assays are rapid and allow reliable toxicity fingerprints for NPs. In the present study, an attempt was made to (1) characterize CeO_2_ NPs to assess their behavior in culture medium, (2) evaluate cytotoxic and oxidative stress potential, (3) estimate their DNA damaging potential and subsequent cell cycle arrest, and (4) evaluate apoptotic index.

## 2. Materials and Methods

### 2.1. Chemicals

Cerium (IV) oxide nanopowder (purity 99.95%, <25 nm particle size; BET), propidium iodide, 2,7-dichlorofluorescein diacetate (DCFDA) dye, 5,5′, 6,6′-tetrachloro-1,1′3′3′-tetraethylbenzimidazolecarbocyanine iodide (JC-1) dye, low melting point agarose (LMA), ethidium bromide (EtBr), Triton X-100, and ethyl methanesulfonate (EMS) were purchased from Sigma Chemical Co. Ltd. (St. Louis, MO, USA). Normal melting agarose (NMA) and ethylenediaminetetraacetic acid (EDTA) disodium salt were purchased from Himedia Pvt. Ltd. (Mumbai, India). Formamidopyrimidine DNA glycosylase (Fpg) enzyme was obtained from Trevigen, Inc. (USA). Hydrogen peroxide (H_2_O_2_) was purchased from Ranbaxy Fine Chemical Ltd. (New Delhi, India). Phosphate buffered saline (Ca^+2^, Mg^+2^ free; PBS), Dulbecco's modified eagle medium : nutrient mixture F-12 (Ham) (1 : 1) powder (DMEM F-12), trypsin-EDTA, foetal bovine serum (FBS), trypan blue, and antibiotic and antimycotic solution (10,000 U/mL penicillin, 10 mg/mL streptomycin, 25 *μ*g/mL amphotericin-B) were purchased from Life Technologies Pvt. Ltd. (New Delhi, India). All other chemicals were obtained locally and were of analytical reagent grade. Cell culture plastic wares were obtained from Thermo Scientific Nunc (Rochester, New York).

### 2.2. Characterization of CeO_2_ NPs

CeO_2_ NPs were characterized using transmission electron microscopy (TEM) and dynamic light scattering (DLS) technique.

#### 2.2.1. Transmission Electron Microscopy (TEM)

TEM analysis was carried out for the assessment of primary particle size and morphology of CeO_2_ NPs. Samples were prepared by suspending NPs in Milli-Q water at a concentration of 50 *μ*g/mL. A drop of suspension was added to the formvar-coated copper grid and allowed to air dry prior to measurement. TEM measurements were performed at an accelerating voltage of 80 kV on a Tecnai G2 spirit (FEI, Netherlands) instrument.

#### 2.2.2. Dynamic Light Scattering (DLS)

CeO_2_ NPs were suspended in Milli-Q water and culture medium (supplemented with 10% FBS) separately at a final concentration of 150 *μ*g/mL and subjected to probe sonication for 10 min at 30 watt for 2 min pulse on and 1 min pulse off cycles. Size and zeta potential of CeO_2_ NPs (100 *μ*g/mL) were analyzed using dynamic light scattering and phase analysis light scattering technique in a Zetasizer Nano-ZS equipped with 4.0 mW, 633 nm laser (model ZEN 3600; Malvern Instruments Ltd., Malvern, UK).

### 2.3. Cell Based Assays

The human lung alveolar basal epithelial cell line (A549) was obtained from National Centre for Cell Science (NCCS), Pune, India, and maintained in DMEM/F-12 medium (1 : 1) supplemented with 10% foetal bovine serum, 1% antibiotic-antimycotic solution at 37°C in a humidified environment of 5% CO_2_. Cells were cultured in 96-well, 12-well, and 6-well culture plates and 75 cm^2^ culture flask for different experiments. Cells at a confluency of 80% were used for all the experiments.

Stock suspension (150 *μ*g/mL) of CeO_2_ NPs was prepared and diluted to varying concentrations (1 *μ*g/mL–100 *μ*g/mL) as described above. Cells were exposed to these concentrations for a specified time schedule (3 h, 6 h, 24 h, and 48 h) as per each experimental design.

#### 2.3.1. Internalization of CeO_2_ NPs

Flow cytometric analysis was performed for the assessment of CeO_2_ NPs internalization in A549 cells according to the method of Suzuki et al. [25] using light scattering principles. The analysis is based on the principle that increase in the intensity of side scattered (SSC) light with constant intensity of forward scattered (FSC) light in cells reveals increased granularity of cells correlated to cellular uptake of NPs. In brief, 1 × 10^5^ cells/mL/well were seeded in 12-well culture plate and allowed to attach the surface for 22 h. Cells were exposed to varying concentrations of CeO_2_ NPs (1 *μ*g/mL–100 *μ*g/mL) for 24 h and 48 h. Treatment was removed and cells were harvested using 0.25% trypsin-EDTA and resuspended in 500 *μ*L of 1x PBS. Analysis was made using flow cytometer equipped with 488 nm laser (FACS Canto II and FACS Diva software (version 6.1.2) BD Biosciences, San Jose, CA, USA).

#### 2.3.2. Cytotoxicity Assessment

Cytotoxicity potential of CeO_2_ NPs was assessed using propidium iodide (PI) staining and trypan blue dye exclusion assay for quantification of dead cells.

*Propidium Iodide (PI) Staining Assay.* Live-dead assessment of A549 cells exposed to CeO_2_ NPs was carried out by flow cytometry based propidium iodide (PI) dye uptake assay.

Cells (1 × 10^5^ cells/mL/well) were seeded in 12-well culture plate and after 22 h exposed to varying concentrations (1 *μ*g/mL–100 *μ*g/mL) of CeO_2_ NPs for 24 h and 48 h. After completion, treated cells were harvested and resuspended in 100 *μ*L PBS and incubated with propidium iodide dye (stock: 5 mg/mL; working: 2 *μ*L/100 *μ*L) for 10 min at room temperature. This suspension was diluted by adding 400 *μ*L PBS and red fluorescence emitted from PI was collected using BD FACS Canto II flow cytometer (BD Biosciences, San Jose, CA, USA) coupled with 650 ± 13 nm band pass filter. The proportions of live or dead cells were analyzed using FACS Diva software (version 6.1.2) (BD Biosciences, San Jose, CA, USA).

*Trypan Blue Dye Exclusion Assay.* Viability of A549 cells exposed to CeO_2_ NPs was determined by trypan blue dye exclusion assay. Briefly, 5 × 10^4^ cells/mL/well were seeded and exposed to different concentrations (1 *μ*g/mL–100 *μ*g/mL) for 24 h and 48 h. Exposed cells were harvested, washed with PBS, and mixed with equal volume (10 *μ*L cells + 10 *μ*L dye) of 0.25% trypan blue dye solution for 5 min. Ten microlitres from this solution were used to count the viable cells by using a Countess automated cell counter (Invitrogen, UK). Loss in viability was expressed as percent dead cells.

### 2.4. Measurement of Oxidative Stress Markers

#### 2.4.1. Determination of Intracellular Reactive Oxygen Species (ROS) Generation

Generation of intracellular reactive oxygen species in A549 cells exposed to CeO_2_ NPs was measured using 2,7-dichlorofluorescein diacetate (DCFDA) dye according to the method of Wang and Joseph [26]. Briefly, 1 × 10^4^ cells/100 *μ*L/well were seeded in 96-well black bottom plate and incubated for 22 h before the exposure. Cells were exposed to increasing concentrations (1 *μ*g/mL–100 *μ*g/mL) of CeO_2_ NPs for 3, 6, and 24 h in the presence and absence of N-acetyl-L-cysteine (NAC) at a concentration of 10 mM. Following the exposure, cells were washed with PBS and incubated with DCFDA dye (20 *μ*M in PBS) for 30 min at 37°C. Further the dye solution was replaced by 200 *μ*L of PBS and fluorescence was read at 485 nm excitation and 528 nm emission wavelength in multiwell plate reader (SYNERGY-HT, Biotek, USA) using KC-4 software. The results were expressed as % ROS generation compared to control. To detect the auto fluorescence and interference of NPs with DCFDA dye, a cell free experiment in presence and absence of CeO_2_ NPs was also conducted in parallel to the treatment experiment.

#### 2.4.2. Measurement of Glutathione (GSH) Level

A549 cells treated with CeO_2_ NPs were collected and assessed for the changes in the level of cellular GSH according to the method of Ellman [27].

Briefly, cells were cultured in 75 cm^2^ flask and exposed to 1 *μ*g/mL–100 *μ*g/mL concentrations of NPs for 3 h and 6 h. After the treatment, cells from control and treated groups were lysed in cell lysis buffer (20 mM Tris-HCl (pH 7.5), 150 mM sodium chloride (NaCl), 1 mM Na_2_EDTA, 1% Triton X-100, and 2.5 mM sodium pyrophosphate). Following centrifugation the cell extract was maintained on ice until assayed for the cellular GSH. A mixture of 0.1 mL cell extract and 0.9 mL of 5% tri-chloro acetic acid (TCA) was centrifuged (2300 ×g for 15 min at 4°C). Further, 0.5 mL of the supernatant was added to 1.5 mL of 0.01% 5,5′-dithiobis(2-nitrobenzoic acid) (DTNB) and the reaction was monitored at 412 nm. The amount of GSH was expressed in terms of umol/mg protein.

### 2.5. Determination of Mitochondrial Membrane Potential and Apoptosis

Determination of mitochondrial membrane potential (MMP) was done using lipophilic cationic 5,5′, 6,6′-tetrachloro-1, 1′3′3′-tetraethylbenzimidazolecarbocyanine iodide (JC-1) dye. This dye has dual fluorescence nature and mitochondrial membrane permeability. This dye passively enters the mitochondria and forms aggregate which gives the red fluorescence. When the potential of mitochondria collapse, this dye can no longer accumulate in the mitochondria and remains in cytoplasm in form of monomer which gives the green fluorescence.

For the MMP analysis, 1 × 10^5^ cells/mL/well cultured in 12-well plate and exposed to 1 *μ*g/mL–100 *μ*g/mL of CeO_2_ NPs for 24 h were harvested and washed with PBS and incubated with 10 *μ*M JC-1 dye in culture medium for 15 min at 37°C. Cells were again washed with PBS and resuspended in 400 *μ*L of PBS. The cells were analyzed for red and green fluorescence in a BD FACS Canto II flow cytometer (BD Biosciences, San Jose, CA, USA) coupled with 485 nm excitation and 590 nm emission filters.

Apoptosis analysis in CeO_2_ NPs exposed A549 cells was carried out using the Annexin V-FITC apoptosis detection kit (BD Biosciences, San Jose, CA, USA) according to the manufacturer's protocol. Briefly, cells (1 × 10^5^ cells/mL/well) exposed to different concentrations (1 *μ*g/mL–100 *μ*g/mL) of CeO_2_ NPs in presence and absence of Z-DEVD-fmk (a Caspase-3 inhibitor) at a concentration of 60 *μ*M for 24 h were harvested, washed with PBS, resuspended in 100 *μ*L of binding buffer containing 5 *μ*L of annexin V and propidium iodide (PI), and incubated for 10 min at room temperature in dark. After incubation, samples were diluted by adding 400 *μ*L of binding buffer and analyzed using BD FACS Canto II flow cytometer equipped with FACS Diva software, version 6.1.2 (BD Biosciences, San Jose, CA, USA). The results were expressed as Annexin V-FITC^+^ and PI^−^ cells identified as apoptotic cells, Annexin V-FITC^+^ and PI^+^ cells as late apoptotic cells, Annexin V-FITC^−^ and PI^+^ cells as necrotic cells, and Annexin V-FITC^−^ and PI^−^ as healthy cells.

Along this, acridine orange staining of A549 cells treated with different concentrations of CeO_2_ NPs was also carried out for the assessment of apoptosis. Briefly, 1 × 10^5^ cells/mL/well were seeded in 12-well plate, exposed to 1 *μ*g/mL–100 *μ*g/mL concentrations of NPs for 24 h, harvested, and cytocentrifuged at 1000 rpm for 10 min. Cells were air dried, fixed in 90% chilled methanol for 10 min, and stained with 10 *μ*g/mL solution of acridine orange dye for 5 min. The visualization of cells and scoring of the slides were done at 400x magnification using fluorescent microscope (DMLB, Leica, Germany) coupled with CCD camera.

### 2.6. Assessment of DNA Damage Induction by CeO_2_ NPs

The DNA damaging potential of CeO_2_ NPs was determined by standard alkaline Comet assay and fpg-modified Comet assay.

#### 2.6.1. Standard Alkaline Comet Assay

The induction of DNA damage by CeO_2_ NPs was assessed by using alkaline Comet assay according to the method of Singh et al. [28]. In brief, 1 × 10^5^ cells/mL/well in 12-well culture plate was exposed to increasing concentrations (1 *μ*g/mL–100 *μ*g/mL) of CeO_2_ NPs for 6 h. Following the exposure, cells were harvested and resuspended in 100 *μ*L PBS. Comet slides were prepared according to the method of Bajpayee et al. [29] and kept in lysis solution at 4°C overnight. Duplicate slides for each concentration were prepared. The slides were subjected to DNA unwinding, electrophoresis, and neutralization process. Slides were stained with 20 *μ*g/mL of ethidium bromide solution and kept in a humidified slide chamber until scoring. The scoring of the slides was done at 400x magnification using fluorescent microscope (DMLB, Leica, Germany) coupled with CCD camera. The analysis was done using image analysis software (KOMET 5.0, Kinetic Imaging, U.K.) attached with microscope. Images from 50 Comet cells (25 cells from each replicate slide) were analyzed and the Comet parameters, that is, % Tail DNA and Olive tail moment, were measured in cells according to the defined protocol [30].

#### 2.6.2. Fpg-Modified Comet Assay

Fpg-modified Comet assay was done according to the method of Collins [31] to identify the oxidative stress mediated DNA damage involving the induction of oxidized bases. Briefly, up to the lysing, the process was performed the same as with standard alkaline Comet assay. After lysing, slides were washed with enzyme buffer (40 mM HEPES, 0.1 M KCl, 0.5 mM EDTA, pH 8; 0.2 mg/mL bovine serum albumin, BSA) for three times and subsequently incubated with 30 *μ*L of fpg enzyme (1 : 3000 dilution in enzyme buffer) for 30 min at 37°C. Further the slides were subjected to unwinding, electrophoresis, staining, and imaging the same as with standard alkaline Comet assay.

### 2.7. Cell Cycle Analysis

It is well known that presence of cells in subG1 phase of cell cycle correlates the DNA fragmentation with apoptosis [32]. Effect of CeO_2_ NPs on cell cycle progression of A549 cells was assessed using Flow cytometry. Briefly, 2 × 10^5^ cells/mL/well in 6-well culture plates were exposed to different concentrations (1 *μ*g/mL–100 *μ*g/mL) of NPs for 24 h. After exposure, cells were harvested, washed with PBS, and fixed with 70% ice cold ethanol overnight at −20°C. Cells were centrifuged, lysed for 30 min at 4°C (using 0.2% Triton X 100), and treated with 10 mg/mL RNase for 30 min at 37°C. Samples were stained with 1 mg/mL solution of propidium iodide dye for 30 min at 4°C and analyzed using BD FACS Canto II flow cytometer equipped with FACS Diva software, version 6.1.2 (BD Biosciences, San Jose, CA, USA). Results were expressed as percentage of cells in each phase of cell cycle.

### 2.8. Western Blotting

For analysis of different proteins involved in CeO_2_ NPs induced toxicity, 2 × 10^5^ cells/mL/well grown in 6-well culture plate were exposed with NPs concentrations (1 *μ*g/mL–100 *μ*g/mL) for 24 h. After completion of exposure, cells were washed three times with PBS and protein was extracted from cells for electrophoresis. The amount of protein was estimated using Bradford method [33] and resolved by SDS-PAGE and transferred to polyvinylidene fluoride (PVDF) membrane. For the detection of specific proteins, PVDF membrane was incubated with anti-BAX, anti-BCl-2, anti-caspase-3, anti-caspase-9, anti-cytochrome C, anti-PARP, anti-p53, and anti-*β*-actin after blocking in 3% BSA solution. Detection of protein bands was carried out using chemiluminiscence and densitometric analysis was done using Quantity One Quantitation Software version 4.3.1 (Bio-Rad, USA).

### 2.9. Statistical Analysis

All the assays were repeated at least three times and the data are presented as mean ± standard error. In all experiments, CeO_2_ NPs treated samples were compared with their respective controls and the mean difference was calculated using one way analysis of variance (ANOVA). Along this, mean difference between two groups was assessed using the two-tailed Student's *t*-test and *P* < 0.05 was considered as statistically significant in all the experiments.

## 3. Results

### 3.1. Characterization of Cerium Oxide Nanoparticles

**Figure 1:**
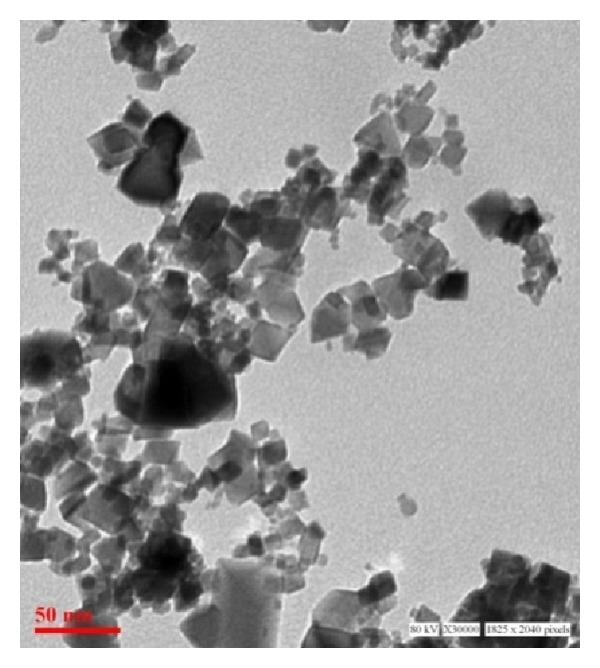
Characterization of CeO_2_ NPs by TEM. TEM analysis revealed that CeO_2_ NPs were cuboidal in shape with size range from ~8 nm to 20 nm with some agglomeration.

TEM analysis was done for the assessment of primary particle size and morphology. CeO_2_ NPs were cuboidal in shape and size observed from TEM was approximately in the range from 8 nm to 20 nm although some agglomeration was also present (Figure 1).

CeO_2_ NPs were also characterized using dynamic light scattering technique in Milli-Q water as well as DMEM F-12 culture medium. In Milli-Q water they tend to form agglomerate with average diameter 576.8 ± 0.45 nm. In DMEM F-12 medium the average size and zeta potential of CeO_2_ NPs was shown to be 177.4 ± 0.23 nm and −13.7 ± 0.25 mV, respectively, with polydispersity index (PDI) 0.248 (Table 1).

### 3.2. Cellular Internalization of Cerium Oxide Nanoparticles

**Figure 2:**
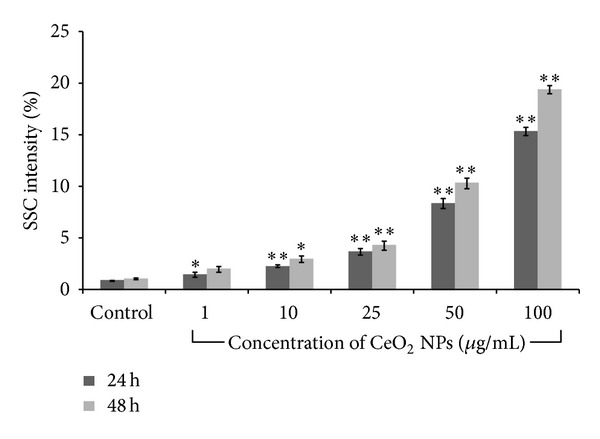
Internalization of CeO_2_ NPs in A549 cells after 24 h and 48 h exposure. Cells were harvested and analyzed using flow cytometry. Increase in SSC intensity which correlates the increased granularity of cells was used as a marker for uptake of NPs. Values represent mean ± SEM of three independent experiments (*P < 0.05, **P < 0.01, compared with control).

There was a statistically significant (*P* < 0.05) concentration and time dependent internalization of nanoparticles as evident by increase in the SSC intensity of treated cells. Result showed that 3.72%, 8.38%, and 15.33% increase in intensity of SSC at 25 *μ*g/mL, 50 *μ*g/mL, and 100 *μ*g/mL, respectively, after 24 h was found which increased to 4.32%, 10.34%, and 19.43% after 48 h (Figure 2).

### 3.3. Morphological Analysis

**Figure 3:**
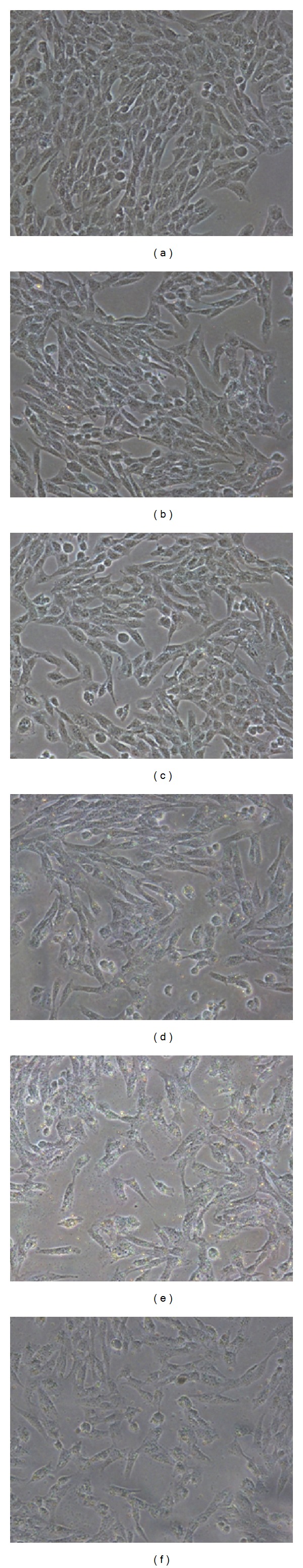
Morphological changes in A549 cells exposed to CeO_2_ NPs for 24 h. Cells were exposed, washed, and examined under phase contrast microscope: (a) control cells, (b) 1 *μ*g/mL, (c) 10 *μ*g/mL, (d) 25 *μ*g/mL, (e) 50 *μ*g/mL, and (f) 100 *μ*g/mL concentration exposed cells (magnification ×20).

Morphological analysis of A549 cells after NPs exposure exhibited that there was an obvious change in cell morphology after 24 h exposure (Figure 3). Cells lost their morphology and started to become round in shape at 25 *μ*g/mL concentration. These changes markedly increased with increasing concentrations and at 100 *μ*g/mL many of the cells detached and formed clumps with irregular shape. Cell density was also reduced at higher dose.

### 3.4. Cytotoxicity Assessment

**Figure 4:**
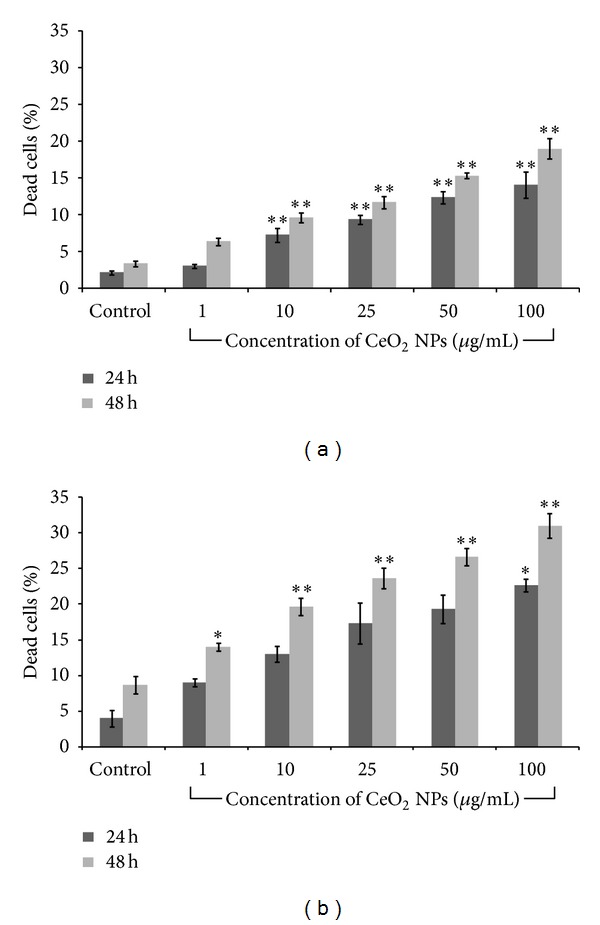
Cytotoxicity assessment of CeO_2_ NPs in A549 cells by (a) propidium iodide (PI) uptake method and (b) trypan blue dye exclusion assay after 24 h and 48 h exposure. Cells were stained with PI and subjected to flow cytometer, whereas in trypan blue assay cells were subjected to automatic cell counter. The results were expressed as mean ± SEM of three independent experiments (*P < 0.05, **P < 0.01, compared with control).

To determine the cytotoxicity, A549 cells were incubated with varying concentrations (1 *μ*g/mL–100 *μ*g/mL) of CeO_2_ NPs for 24 h and 48 h. Propidium iodide (PI) uptake method was utilized to assess the viability in terms of cell death. Cell death was increased in a concentration and time dependent manner following the exposure of nanoparticles (Figure 4(a)).

There was a statistically significant (*P* < 0.01) increase in cell death (7.25%, 9.37%, 12.35%, and 14.07% in 10 *μ*g/mL, 25 g/mL, 50 *μ*g/mL, and 100 *μ*g/mL exposed cells, resp.) after 24 h exposure which increased to 9.62%, 11.69%, 15.34%, and 18.98%, respectively, after 48 h exposure. Similar results were also obtained by trypan blue assay in which viability of cells was decreased with increasing concentrations of NPs (Figure 4(b)).

### 3.5. Determination of ROS and GSH Amount

**Figure 5:**
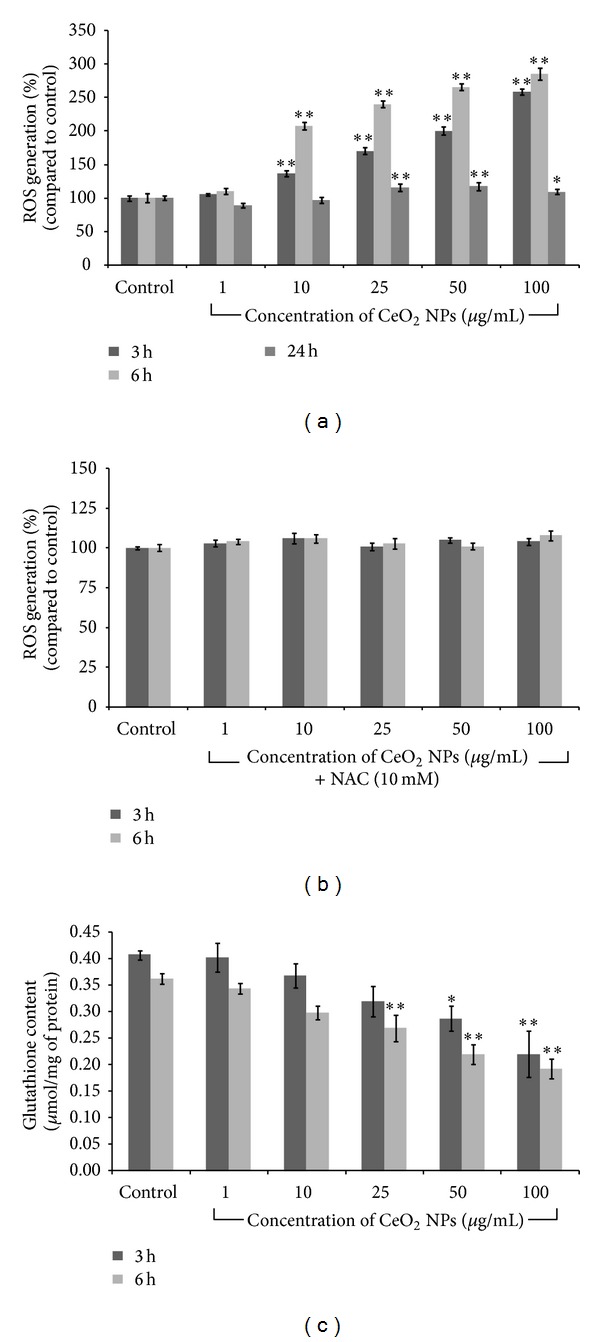
Effect of CeO_2_ NPs on (a, b) ROS production and (c) cellular level of glutathione (GSH) in A549 cells. (a, b) Cells were exposed and incubated with H_2_DCFDA. (c) After exposure cells were collected, lysed, and incubated with reaction mixture (TCA, DTNB). Both measurements were done using microplate reader and data represents mean ± SEM of three independent experiments (*P < 0.05, **P < 0.01 compared to control).

CeO_2_ NPs showed a significant (*P* < 0.01) concentration and time dependent increase in production of ROS in terms of increase in DCF fluorescence intensity (Figure 5(a)). DCF fluorescence intensity increased to 171%, 200%, and 259% after 3 h and 240%, 266%, and 286% after 6 h exposure of 25 *μ*g/mL, 50 *μ*g/mL, and 100 *μ*g/mL, respectively, as compared to control. However, ROS generation decreased (115%, 118%, and 109% at 25 *μ*g/mL, 50 *μ*g/mL, and 100 *μ*g/mL) after 24 h exposure. This ROS was completely reduced in the presence of N-acetyl-L-cysteine (NAC) as shown in Figure 5(b).

With the increase in ROS production cellular GSH significantly (*P* < 0.05) depleted after 3 h and 6 h at 25 *μ*g/mL, 50 *μ*g/mL, and 100 *μ*g/mL exposure as compared to control (Figure 5(c)).

### 3.6. Mitochondrial Membrane Potential (MMP) Analysis and Apoptosis

**Figure 6:**
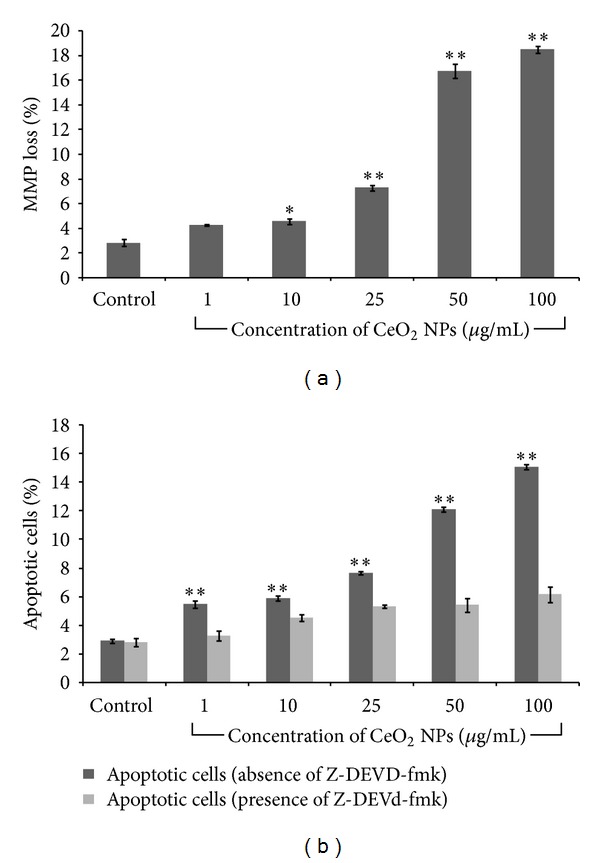
Mitochondrial membrane potential alteration and apoptosis induction by CeO_2_ NPs in A549 cells after 24 h exposure. Cells were exposed to 1 *μ*g/mL–100 *μ*g/mL concentration of NPs, harvested, and stained with JC-1 dye for MMP (Figure 6(a)) and annexin V-FITC/PI for apoptosis (Figure 6(b)). Cells were subjected to flow cytometry and values from three independent experiments (mean ± SEM) were reported (*P < 0.05, **P < 0.01 compared to control).

Cells treated with CeO_2_ NPs showed a significant (*P* < 0.05) concentration dependent decrease in mitochondrial membrane polarization as evident by JC-1 dye (Figure 6(a)). There was 7.27%, 16.76%, and 18.51% decrease in MMP at 25 *μ*g/mL, 50 *μ*g/mL, and 100 *μ*g/mL exposure, respectively, as compared to control.

Both the decrease in MMP and accumulation of ROS are hallmarks of mitochondria mediated apoptosis [34]. As evident in Figure 6(b), the amount of apoptotic cells also increased in concentration dependent manner. There was increase in apoptotic cells from 2.79% (control) to 6.79% (25 *μ*g/mL), 7.37% (50 *μ*g/mL), and 8.92% (100 *μ*g/mL). However, in the presence of Z-DEVD-fmk, this death was completely attenuated which clearly indicated that the death was caspase dependent.

### 3.7. Acridine Orange Staining

**Figure 7:**
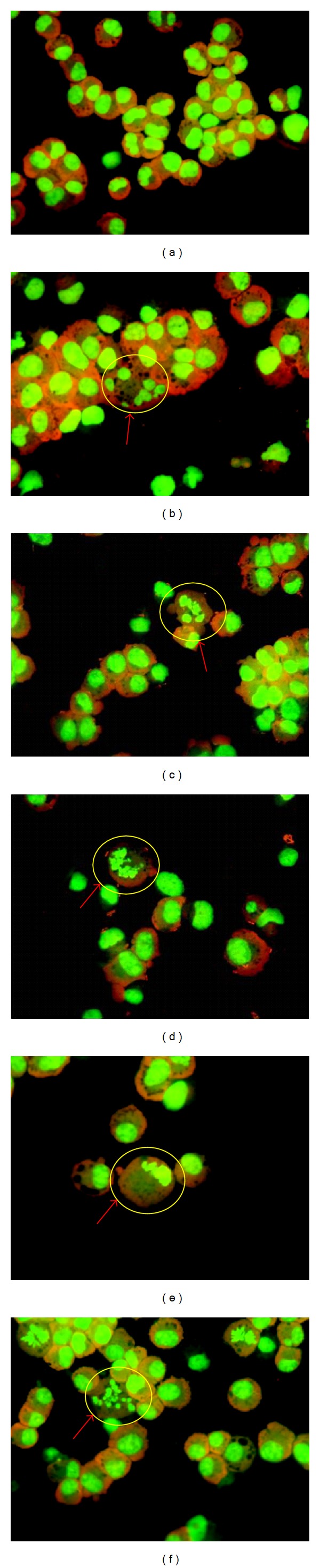
Determination of apoptotic cells by acridine orange staining of A549 cells exposed to CeO_2_ NPs for 24 h exposure. Cells were exposed, washed, and stained with acridine orange dye. (a) Control cells, (b, c) treated cells showing nuclear fragmentation, (d, e) chromatin condensation, and (f) apoptotic bodies.

Determination of apoptosis induction in A549 cells by CeO_2_ NPs was also assessed by acridine orange staining. Cells with chromatin condensation, nuclear fragmentation, and apoptotic bodies were found in the cells exposed to CeO_2_ NPs as compared to control (Figure 7).

### 3.8. DNA Damage by CeO_2_ NPs

Cells exposed to 25 *μ*g/mL, 50 *μ*g/mL, and 100 *μ*g/mL concentration of CeO_2_ NPs for 6 h exhibited a significant (*P* < 0.05) induction in DNA damage compared to control cells as evident by Comet parameters, Olive tail moment, and % Tail DNA in standard alkaline Comet assay (Table 2). Moreover, the values for % Tail DNA and Olive tail moment in fpg-modified Comet assay were significantly (*P* < 0.05) higher than the standard alkaline Comet assay. This result suggests the involvement of oxidative stress towards the DNA damage.

### 3.9. Cell Cycle Analysis

**Figure 8:**
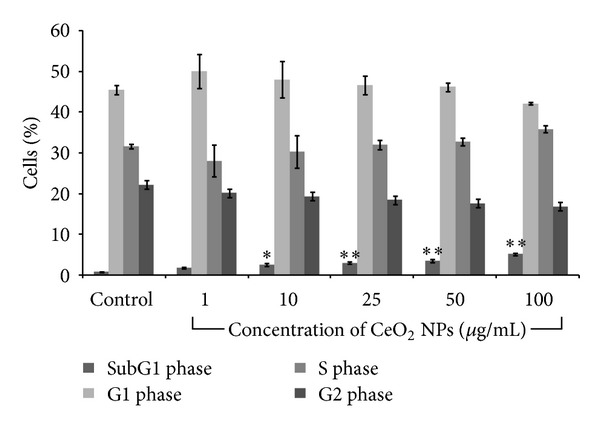
Cell cycle analysis of A549 cells exposed to CeO_2_ NPs for 24 h exposure. Cells were exposed, fixed, and stained with propidium iodide dye before subjecting to flow cytometric analysis. Values from three independent experiments (mean ± SEM) were reported where *P < 0.05 and **P < 0.01 compared to control.

The effect of NPs on A549 Cell cycle progression was assessed by flow cytometry. In response to DNA damage, relevant checkpoints can arrest the cell cycle at a certain stage. Data showed that there was a significant (*P* < 0.05) dose dependent increase in cells present in subG1 phase of cell cycle in comparison to control while the percentage of cells in G2 phase of cell cycle declined progressively. The amount of cells in Sub G1 phase increased from 0.7% (control) to 2.95%, 3.45%, and 5.1% at 25 *μ*g/mL, 50 *μ*g/mL, and 100 *μ*g/mL concentration, respectively (Figure 8).

### 3.10. Western Blot Analysis

**Figure 9:**
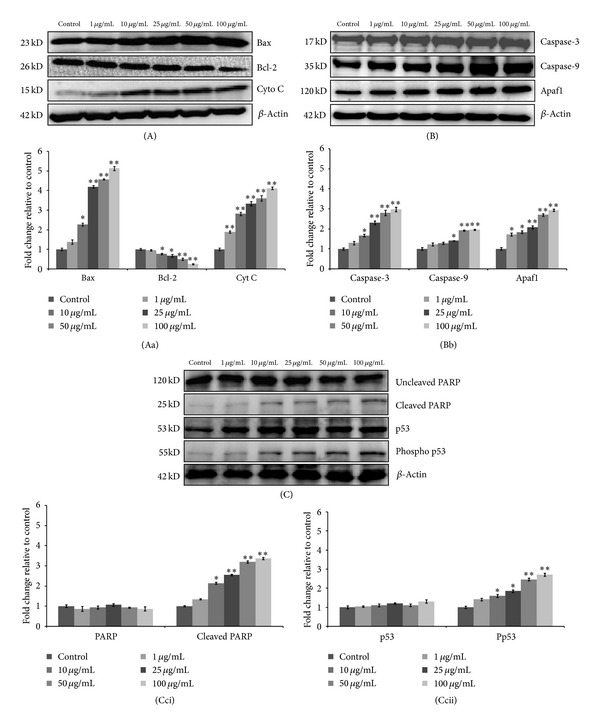
Dose dependent expression level analysis (A, B, and C) of various apoptotic proteins in A549 cells after 24 h exposure of CeO_2_ NPs. Cells exposed to indicated concentration, protein lysate, were collected and assayed by western blotting. *β*-actin was used as an internal control. All blots (A, B, and C) and respective Bar graph (Aa, Bb, and Cc) values (mean ± SEM) are representative of three independent experiments (*P < 0.05, **P < 0.01 compared to respective control).

Western blot analysis data exhibited that there was a significant (*P* < 0.05) increase in expression of proapoptotic protein Bax (4.1 fold, 4.5 fold, 5.1 fold), tumor suppressor protein p53 (1.8-fold, 2.4-fold, and 2.7-fold), and PARP (2.5-fold, 3.1-fold, and 3.3-fold) with decreased expression level of Bcl-2 (0.66-fold, 0.49-fold, and 0.23-fold) as compared to control at 25 *μ*g/mL, 50 *μ*g/mL, and 100 *μ*g/mL concentrations, respectively. Furthermore, significant (*P* < 0.05) increase in expression level of cytochrome C (3.3-fold, 3.6-fold, and 4.1-fold), Apaf-1 (2.0-fold, 2.6-fold, and 2.9-fold), caspase-3 (2.2-fold, 2.7-fold, 2.9-fold), and caspase-9 (1.4-fold, 1.9-fold, and 2-fold) were also found which confirms the mitochondrial mediated apoptotic cell death induction by CeO_2_ NPs in A549 cell (Figure 9).

## 4. Discussion

Due to the conflicting information of cerium oxide nanoparticles effects over human and environment, the present study was carried out to precisely define their final fate in a cell. Moreover, the major regulator behind the CeO_2_ NPs induced cell death, if any, was also explored. Our study systematically concludes that CeO_2_ NPs get internalized in cells in a significant manner and lead to mitochondria mediated caspase dependent cell death. We also showed that CeO_2_ NPs produced increased amount of ROS which contributed to extensive DNA damage and perturbation in cell cycle with increasing apoptotic cells in subG1 phase. Further, in the presence of specific inhibitor for ROS and apoptosis, both processes were completely attenuated, proving that the cause of CeO_2_ toxicity is ROS mediated DNA damage leading to apoptosis.

The characterization of NPs is essential for the toxicity studies as it has been shown that the size, shape, surface reactivity, solubility, and degree of aggregation impart for their differential response in biological system [35]. Hence, the particle morphology, average size, and agglomeration status of CeO_2_ were examined by TEM. We observed that the particles were in the range of 8 nm to 20 nm in size and cuboidal in shape, although there was some agglomeration also present which range in the size of ~40 nm. Also, to mimic the real exposure scenario the CeO_2_ NPs were characterized in the Milli-Q water as well as in culture medium by DLS. We observed that the average hydrodynamic size of the particle in Milli-Q water was 576.8 nm ± 0.45 nm and in culture medium 177.4 nm ± 0.23 nm. The difference in the average hydrodynamic size of NPs observed in different solvent could be attributed to their aggregation state. In the culture media NPs were more stable and monodispersed due to the formation of protein nanoparticles corona (Table 1).

For toxicity studies of the NPs, it is important to assess NPs uptake and correlate it with the cellular response. In the present study the internalization of the CeO_2_ NPs in A549 cells were determined by the flow cytometry using the protocol described by our earlier studies [36, 37]. We observed a concentration and time dependent significant (*P* < 0.05) increase in the side scattered intensity of the treated A549 cells as compared to control (Figure 2). Additionally, phase contrast images of A549 cells treated with CeO_2_ NPs showed their accumulation in cytoplasm. The uptake potential of CeO_2_ NPs in various human cell lines as well as in mouse macrophage cells has also been reported previously [38, 39] which correlates with our findings. Also, we observed some morphological changes in the CeO_2_ NPs treated cells such as at concentration 25 *μ*g/mL few cells become spherical while at concentration 100 *μ*g/mL the number of the spherical cells increased and many of them were detached from the surface of culture plate (Figure 3). Earlier studies have also shown the internalization of CeO_2_ NPs in human keratinocytes cells, human bronchial epithelial cells, cardiac progenitor cells, human monocytes, and so forth, using TEM and flow cytometry [40–42].

Various metal oxide nanoparticles have shown to induce cell death after internalization [37, 43, 44]. We assessed the cytotoxicity potential of CeO_2_ NPs using propidium iodide (PI) uptake method and trypan blue dye exclusion assay. PI dye selectively enters the cells with compromised membrane integrity and binds to the DNA which causes increase in the fluorescence intensity of PI dye, whereas in trypan blue dye exclusion assay live cell excludes the dye and dead cells stained with blue color. Our data suggested that following the exposure of CeO_2_ NPs there was a significant (*P* < 0.05) concentration and time dependent cytotoxicity in A549 cells at 25 *μ*g/mL, 50 *μ*g/mL, and 100 *μ*g/mL concentrations at 24 h and 48 h in both the assays. These results were in accordance with the earlier studies which have reported the toxic behavior of CeO_2_ NPs in various cell lines [17, 41]. Cell death induction by various metal oxide NPs are due to their dissolution in culture medium which causes release of ions. These ions contributed to the cell death [43, 45, 46]. But CeO_2_ NPs did not dissolve in medium [47] so that their toxicity is due to NPs and not by ceric ions, although data are also available which show that CeO_2_ NPs exposure did not induce cell death in lung cells and phagocytic cells [46].

NPs mediated cell death is supposed to be due to the production of ROS leading to oxidative stress caused by nanoparticles' small size and large surface area to volume ratio [48, 49]. Our result showed that CeO_2_ NPs are capable to produce ROS in a concentration and time dependent manner up to 6 h (Figure 5(a)). But level of ROS was found to be decreased at 24 h. The possible reasons may be either due to the increase in amount of cell death or generation of ROS stabilized after a certain time period. However, the amount of ROS was also decreased in the presence of NAC, a ROS inhibitor, as shown in Figure 5(b). Further with the increase in amount of ROS, level of GSH was decreased in present study. It is well known that excess production of ROS leads to decreased level of antioxidant in cultured cells. Thus, combined data showed that CeO_2_ NPs are capable to produce ROS which is due to decrease in level of cellular antioxidants which are correlated with previous studies [16]. CeO_2_ NPs have also been reported to show antioxidant behavior [15] which has been attributed to the presence of mixed valence states of CeO_2_ NPs on their surface [9, 50]. However, the exact mechanism of oxidant/antioxidant behavior of CeO_2_ NPs remains elusive.

Any imbalance between ROS level and antioxidant level may lead to genomic instability. So we assessed the effect of CeO_2_ NPs induced ROS on genomic DNA by alkaline Comet assay and fpg modified Comet assay. It was found that the amount of DNA damage was increased in concentration and time dependent manner as suggested by Comet assay parameters, namely, Olive tail moment and Tail DNA (Table 2). The amount of DNA damage was higher in fpg modified Comet assay in comparison to alkaline Comet assay proving the oxidative stress induced DNA damage. In earlier studies it has been reported that CeO_2_ NPs did not induce damage to DNA and chromatin in human cells [51] and have antioxidative effect over trichloroethane (TCEtn) exposure [52] which are in contrary to our results. But in these studies cell lines were of different origins and in the earlier study [52] effect of cerium oxide alone has not been shown.

Excess production of ROS with concomitant decrease in GSH level also leads to mitochondrial membrane permeability and induction of apoptotic cell death in cultured cells [53–55]. MMP is important to maintain the potential difference across the mitochondrial membrane. Loss in MMP is an early event during apoptosis in cultured cells which results in accumulation of ROS and redistribution of apoptotic factors across the mitochondria [56]. In present study we assessed the MMP loss by the use of JC-1 dye which has dual color properties. In healthy cells, this dye enters the mitochondria and forms aggregate which gives red color but when the potentials of mitochondria collapse, this dye remains in cytoplasm and gives green color. Thus, increase in red to green fluorescence ratio was used as a marker of MMP loss. Our results showed that there was a significant (*P* < 0.05) concentration dependent decrease in mitochondrial membrane potential. Increased production of ROS with progressive decrease in MMP has been proposed as main event during the apoptosis [34]. Here we assessed the apoptosis induction by CeO_2_ NPs using annexin V-FITC/PI. We found that there was a concentration dependent increase in apoptotic cells which were reversed in the presence of Z-DEVD-fmk. However, necrotic and late apoptotic cells were not found. Moreover, in cell cycle analysis, we found the significant increase in amount of cell present in sub G1 phase as compared to control.

Further, to understand the molecular mechanism behind the apoptotic cell death induced by CeO_2_ NPs in A549 cells Western blot analysis was carried out after 24 h exposure. Our results showed that there was a concentration dependent increase in p53 level of treated cells as compared to control cells (Figure 9(C)). Expression of p53 proteins in mammals is tightly regulated and serves to protect the organism from DNA-damaging stimuli. In case of extensive DNA damage, p53 activates and leads to cell cycle arrest or apoptosis [57]. It has also been shown that activation of p53 leads to upregulation of proapoptotic member of Bcl-2 family such as Bax and Bak which translocates to mitochondria and suppresses the activity of antiapoptotic member of Bcl-2 family [58]. This modulation in Bax/Bcl-2 ratio leads to permeabilization of mitochondria and release of apoptotic proteins such as cytochrome C which binds to cytosolic Apaf-1 [59]. This binding results in oligomerization of Apaf-1 and this complex further binds to caspase-9 to form apoptosome which cleaves and activates the downstream caspases (3, 6, and 7) to initiate mitochondrial mediated apoptosis [60]. In addition to this our data also exhibited that there was a concentration dependent increase in Bax, cytochrome C, caspase-9, and Apaf-1 with progressive decrease in Bcl-2 protein expression which suggest the induction of apoptotic cell death pathway in A549 cells exposed to CeO_2_ NPs (Figures 9(A) and 9(B)). Consequently there was an increase in expression level of caspase-3 along with PARP, a downstream substrate of activated caspase-3, which confirms the involvement of mitochondria in apoptotic cell death.

## 5. Conclusion

Present study showed that cerium oxide NPs get internalized in A549 cells which cause morphological alterations and toxicity in a concentration and time dependent manner. These NPs increase the production of ROS which leads to decrease in antioxidant level of cells and apoptotic cell death. Along with this, ROS production also causes damage to DNA and halts cell cycle progression which contributed to increased amount of cell death. Immunoblot analysis exhibited that the apoptotic death was mediated through mitochondria which involves the activation of Bcl-2 family mediator including activation of PARP. Thus, extensive DNA damage mediated molecular perturbations leads to apoptotic cell death in CeO_2_ NPs exposed A549 cells.

## Supplementary Material

Graphical diagram elucidating the mechanism of CeO_2_ induced toxicity in A549 cells.

## Figures and Tables

**Table 1:** Characterization of CeO_2_ NPs by dynamic light scattering.

S. number	Medium	Hydrodynamic size (d.nm)	Polydispersity index (PDI)	Zeta potential (mV)
1	Milli-Q water	576.8	0.862	−11.9
2	Culture medium (DMEM F-12 with 10% FBS)	177.4	0.248	−13.7

**Table 2:** DNA damage induction by CeO_2_ NPs as evident by alkaline and fpg modified Comet assay in A549 cells after 6 h exposure.

Groups	OTM (arbitrary unit)		Tail DNA (%)	
	Fpg (−)	Fpg (+)	Fpg (−)	Fpg (+)
Control	0.89 ± 0.06	1.13 ± 0.09	6.32 ± 0.23	6.96 ± 0.12
CeO_2_ NPs (1 *μ*g/mL)	1.19 ± 0.12*	1.68 ± 0.14^∗#^	7.76 ± 0.38	10.81 ± 0.28^∗∗#^
CeO_2_ NPs (10 *μ*g/mL)	1.55 ± 0.07**	1.94 ± 0.15^∗∗#^	9.01 ± 0.22*	11.82 ± 0.24^∗∗#^
CeO_2_ NPs (25 *μ*g/mL)	1.73 ± 0.08**	2.28 ± 0.15^∗∗#^	10.30 ± 0.67**	13.45 ± 0.18^∗∗#^
CeO_2_ NPs (50 *μ*g/mL)	1.89 ± 0.07**	2.70 ± 0.14^∗∗#^	13.15 ± 0.61**	15.63 ± 0.26^∗∗#^
CeO_2_ NPs (100 *μ*g/mL)	2.13 ± 0.07**	3.76 ± 0.16^∗∗#^	16.39 ± 0.65**	18.04 ± 0.27^∗∗#^
Positive control-H_2_O_2 _(25 *μ*M)	4.39 ± 0.11**	6.33 ± 0.14^∗∗#^	23.67 ± 1.58**	29.28 ± 0.36^∗∗#^

Comet slides were prepared according to defined protocol and 50 Comet cells were scored using fluorescence microscope. Results from three independent experiments in form of mean ± SEM were reported (*P < 0.05, **P < 0.01 compared to respective control; ^#^ *P* < 0.05 when compared with respective values in standard Comet assay).
