# Corrigendum to “Cerium Oxide Nanoparticles Induced Toxicity in Human Lung Cells: Role of ROS Mediated DNA Damage and Apoptosis”

**DOI:** 10.1155/2018/6349540

**Published:** 2018-12-16

**Authors:** Sandeep Mittal, Alok K. Pandey

**Affiliations:** ^1^Academy of Scientific and Innovative Research (AcSIR), New Delhi 110025, India; ^2^Nanomaterial Toxicology Group, CSIR-Indian Institute of Toxicology Research (CSIR-IITR), P.O. Box 80, Mahatma Gandhi Marg, Lucknow, Uttar Pradesh 226001, India

In the article titled “Cerium Oxide Nanoparticles Induced Toxicity in Human Lung Cells: Role of ROS Mediated DNA Damage and Apoptosis” [1], there was figure duplication in Figure 9(c), where the second panel, Cleaved PARP, is similar to the fourth one, Phosopho p53. Figure 9 including the revised image for panel four, Phosopho p53, is shown below and the original figures are added as supplementary materials (available here).

## Supplementary Materials

Supplementary Materials The original uncropped and unadjusted Western Blots for all the panels in Figure 9a, 9b, and 9c.

## Figures and Tables

**Figure 9:**
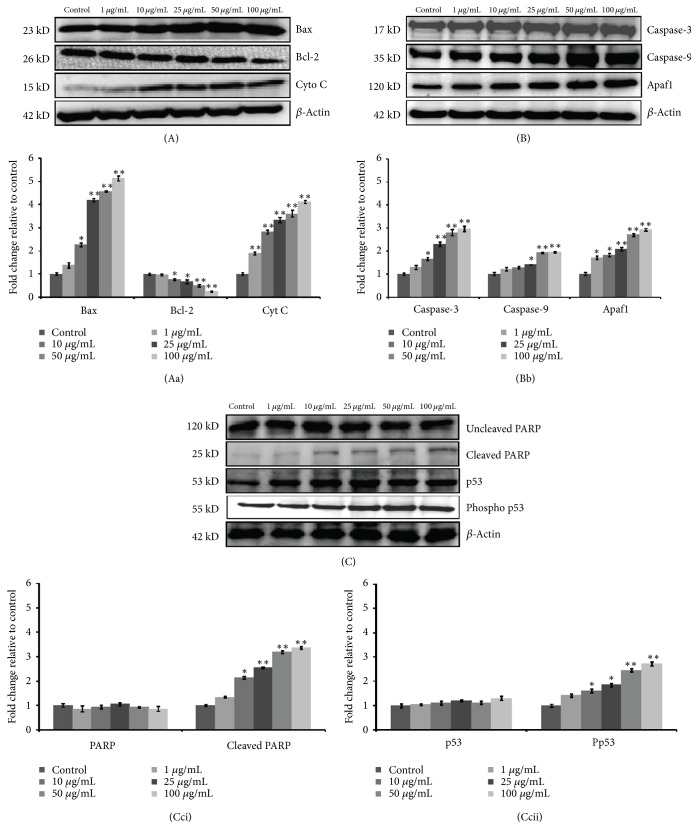
Dose dependent expression level analysis (A, B, and C) of various apoptotic proteins in A549 cells after 24 h exposure of CeO2 NPs. Cells exposed to indicated concentration, protein lysate, were collected and assayed by western blotting. *β*-actin was used as an internal control. All blots (A, B, and C) and respective Bar graph (Aa, Bb, and Cc) values (mean ± SEM) are representative of three independent experiments (*∗P* < 0.05, *∗∗P* < 0.01 compared to respective control).
