# Supplementary material for: Cerium Oxide Nanoparticles Induced Toxicity in Human Lung Cells: Role of ROS Mediated DNA Damage and Apoptosis
Source: Biomed Res Int. 2014 Jun 1;2014:891934. doi: 10.1155/2014/891934 (PMC4058670; doi:10.1155/2014/891934)
Supplement: Supplementary file 1 — Graphical diagram elucidating the mechanism of CeO2 induced toxicity in A549 cells. [file 891934.f1.pdf]

Graphical Abstract:

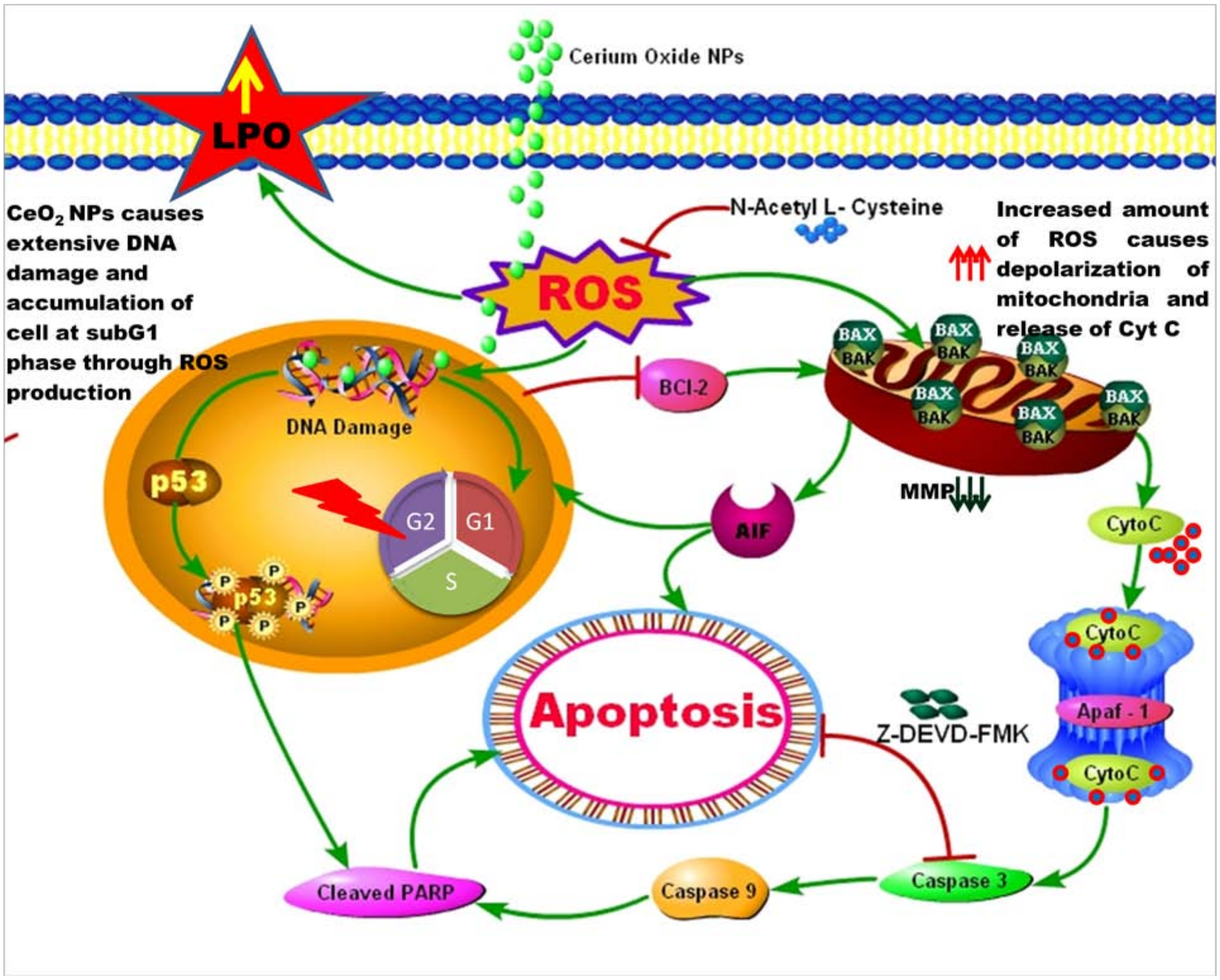

Graphical diagram elucidating the mechanism of CeO_2 induced toxicity in A549 cells
