# Supplementary material for: Corrigendum to “Cerium Oxide Nanoparticles Induced Toxicity in Human Lung Cells: Role of ROS Mediated DNA Damage and Apoptosis”
Source: Biomed Res Int. 2018 Dec 16;2018:6349540. doi: 10.1155/2018/6349540 (PMC6311285; doi:10.1155/2018/6349540)

Panel – 1 (Figure 9A)

BAX

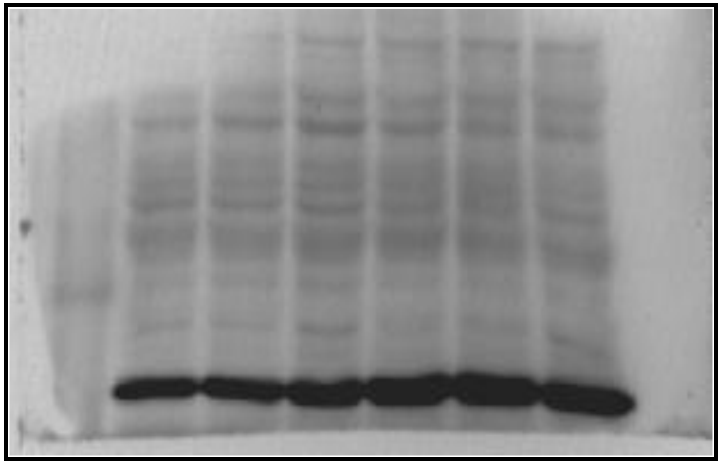

BCl-2

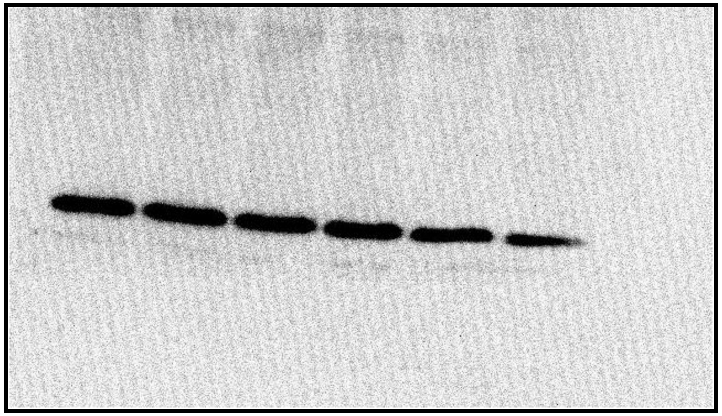

Cyto C

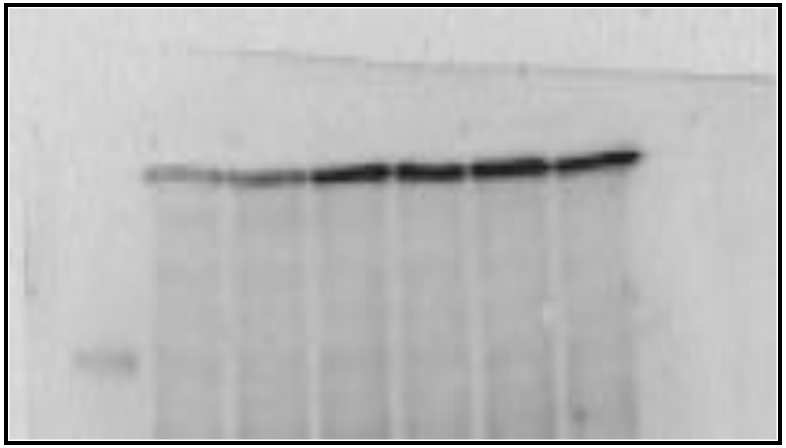

β-Actin

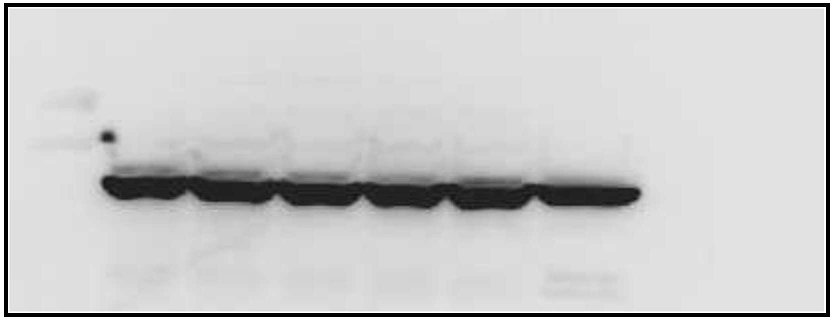

Panel – 2 (Figure 9B)

Caspase 3

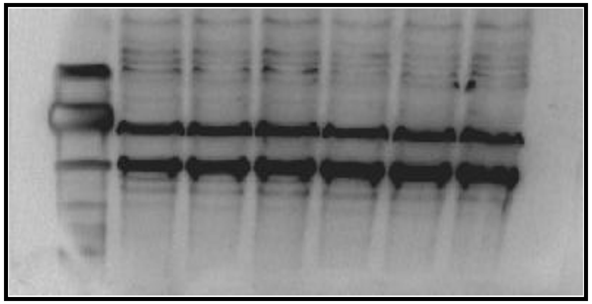

Caspase 9

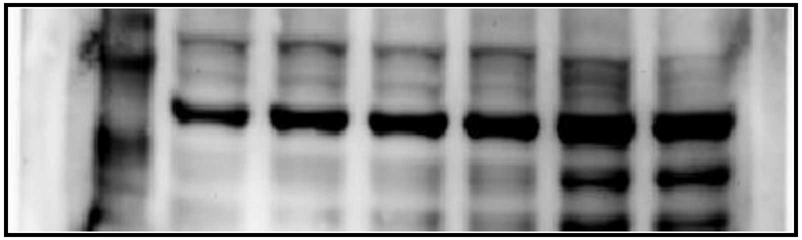

Apaf-1

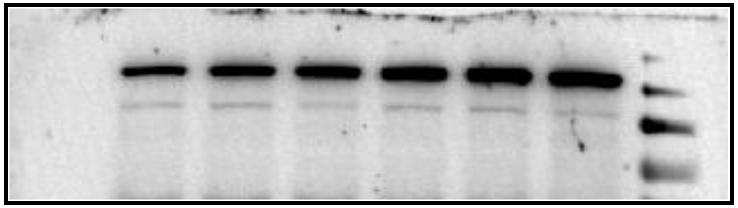

β-Actin

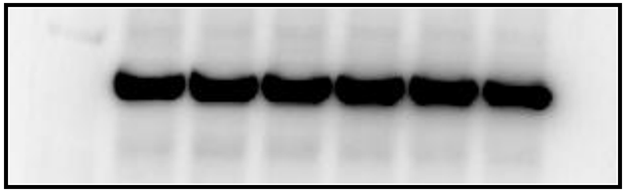

Panel – 3 (Figure 9C)

Uncleaved PARP

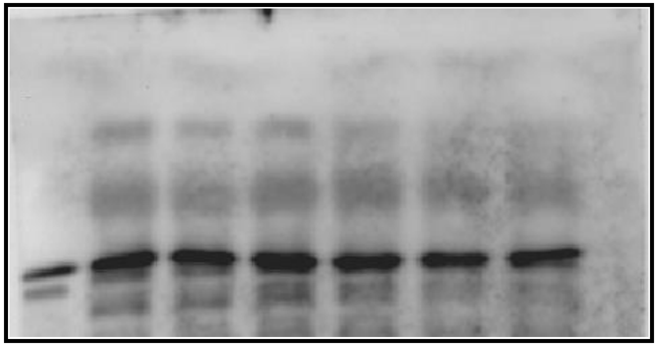

p53

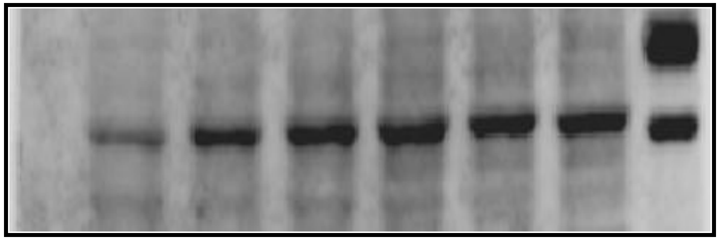

PARP

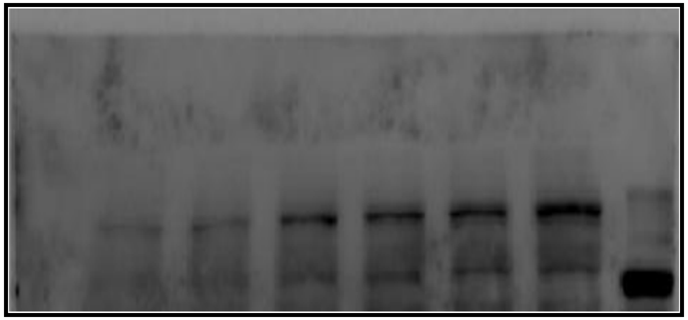

β-Actin

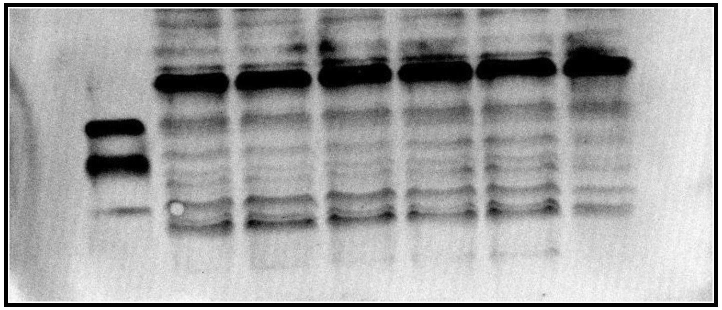

Revised Image for Panel – 3 (Figure 9C)

Phospho p53

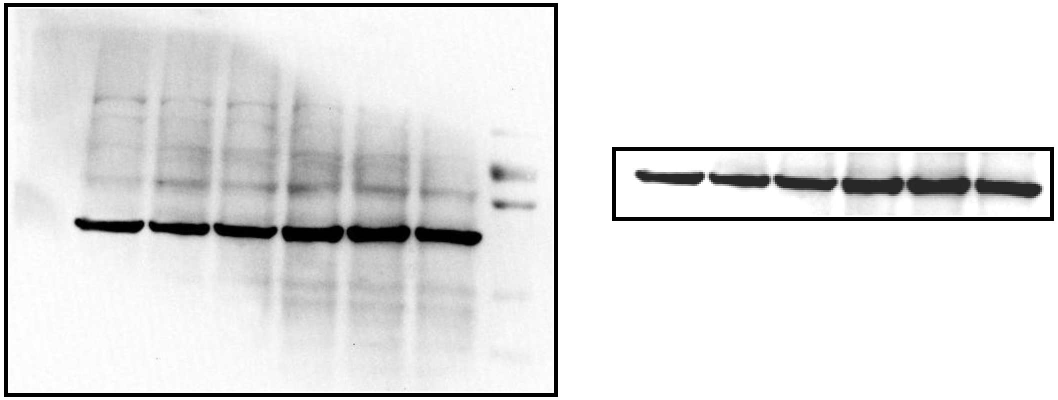

Supplement: Supplementary Materials — The original uncropped and unadjusted Western Blots for all the panels in Figure 9a, 9b, and 9c. [file 6349540.f1.pdf]
